# Genome-wide identification of the geranylgeranyl pyrophosphate synthase (GGPS) gene family involved in chlorophyll synthesis in cotton

**DOI:** 10.1186/s12864-023-09249-w

**Published:** 2023-04-05

**Authors:** Wenxiang Feng, Teame Gereziher Mehari, Hui Fang, Meijun Ji, Zijian Qu, Mengxue Jia, Dongmei Wang, Allah Ditta, Muhammad K. R. Khan, Yunying Cao, Jianyong Wu, Baohua Wang

**Affiliations:** 1 School of Life Sciences, Nantong University, Nantong, Jiangsu 226019 China; 2 State Key Laboratory of Cotton Biology, Institute of Cotton Research of Chinese Academy of Agricultural Sciences, Anyang, Henan 455000 China; 3 Plant Breeding and Genetics Division, Nuclear Institute for Agriculture and Biology, Faisalabad, 38000 Pakistan

**Keywords:** Cotton, Geranylgeranyl pyrophosphate synthase, Virus-induced gene silencing, *Gohir.A13G151300*, Chlorophyll content

## Abstract

**Background:**

Geranylgeranyl pyrophosphate synthase (GGPS) is a structural enzyme of the terpene biosynthesis pathway that is involved in regulating plant photosynthesis, growth and development, but this gene family has not been systematically studied in cotton.

**Results:**

In the current research, genome-wide identification was performed, and a total of 75 GGPS family members were found in four cotton species, *Gossypium hirsutum*, *Gossypium barbadense*, *Gossypium arboreum* and *Gossypium raimondii*. The GGPS genes were divided into three subgroups by evolutionary analysis. Subcellular localization prediction showed that they were mainly located in chloroplasts and plastids. The closely related GGPS contains a similar gene structure and conserved motif, but some genes are quite different, resulting in functional differentiation. Chromosome location analysis, collinearity and selection pressure analysis showed that many fragment duplication events occurred in GGPS genes. Three-dimensional structure analysis and conservative sequence analysis showed that the members of the GGPS family contained a large number of α-helices and random crimps, and all contained two aspartic acid-rich domains, DDxxxxD and DDxxD (x is an arbitrary amino acid), suggesting its key role in function. Cis-regulatory element analysis showed that cotton GGPS may be involved in light response, abiotic stress and other processes. A GGPS gene was silenced successfully by virus-induced gene silencing (VIGS), and it was found that the chlorophyll content in cotton leaves decreased significantly, suggesting that the gene plays an important role in plant photosynthesis.

**Conclusions:**

In total, 75 genes were identified in four *Gossypium* species by a series of bioinformatics analysis. Gene silencing from GGPS members of *G. hirsutum* revealed that GGPS plays an important regulatory role in photosynthesis. This study provides a theoretical basis for the biological function of GGPS in cotton growth and development.

**Supplementary Information:**

The online version contains supplementary material available at 10.1186/s12864-023-09249-w.

## Background

Geranylgeranyl pyrophosphate synthase (GGPS) is a structural enzyme in the terpene biosynthesis pathway and a member of the isopentenyl pyrophosphate synthase gene family. Terpenoids are the largest and most diverse plant-specific metabolites and play important biological roles in various physiological processes, such as growth, photosynthesis, signal transduction, environmental adaptation and stress tolerance, during plant development [1]. All terpenoids are derived from the basic unit structure of five carbon atoms: isopentenyl pyrophosphate (IPP) and its allyl isomer dimethyl allyl pyrophosphate (DMAPP). In the plastids of plants, IPP and DMAPP are synthesized by the 2-C-methyl-D-erythritol-4-phosphate (MEP) pathway. Three molecules of IPP and one molecule of DMAPP form the 20-carbon compound geranylgeranyl pyrophosphate (GGPP) in the action of GGPS. GGPP continues to be catalyzed to form diterpenes and tetri-terpenes [2]. GGPP is not only the precursor of diterpenoids and carotenoids but also the common precursor of tocopherol, abscisic acid, gibberellin, quinone and other polyterpenes. It is the node of many important secondary metabolic pathways in plants [3]. GGPS is a key enzyme in the synthesis of octahydrolycopene in carotenoids [4]. Carotenoid is a fat-soluble pigment that is often located in the chloroplast and chromoplast membranes. Carotenoids can protect chlorophyll from photooxidation damage caused by strong light, and they are an indispensable structural component of the photosynthetic antenna and reaction center complex. In addition, carotenoids are an important component of some pigment-protein complexes [5] and are the precursor of abscisic acid (ABA) [6]. The GGPS gene was first isolated from pepper [7] and then isolated from tomato, Salvia miltiorrhiza, tobacco, Ginkgo biloba and other plants. In relation to the role of glucose, the enzyme UDP-glucose pyrophosphorylase (UGP), a member of the glycosyltransferase gene family, catalyzes the reaction between glucose-1-phosphate and UTP to produce uridine diphosphate glucose (UDPG). Different roles are played by UGP genes. UGP is a necessary substance for b-1,3 glucan and b-1,6 glucan both of which are basic building blocks for the biosynthesis of the cell wall in fungi in the formation of UDPG [8].

In recent years, some progress has been made in the functional research of GGPS genes. The physiological and biochemical functions of GGPS are closely related to its tissue expression characteristics and subcellular localization. There are 12 GGPS genes in the *Arabidopsis thaliana* genome [9]. Different family members are responsible for the synthesis of GGPP in different subcellular structures, in which *AtGGPS1* and *AtGGPS3* are located in plastids, *AtGGPS2* and *AtGGPS4* are distributed in mitochondria, and *AtGGPS6* is located in the endoplasmic reticulum [10]. GGPS1, located in mitochondria, uses GPP to synthesize gibberellins and GGPS11, which are located in plastids and are the core of photosynthesis. GGPP is widely used in the synthesis of chlorophylls, carotenoids and other compounds [11]. Two GGPS genes have been identified in tomato, and the two genes were found in all tissues and organs. In sweet potato, overexpression of the *IbGGPS* gene can upregulate genes related to the glycolysis pathway, MEP pathway and carotenoid pathway and increase the content of carotenoids in transgenic plants. These results suggested that the *IbGGPS* gene has the potential to increase the content of carotenoids in sweet potato and other plants [12]. In addition, *LeGGPS1* expression can be induced when plants are subjected to biological stress [13]. The specifically expressed GGPS genes in flowers and fruits are involved in the synthesis of carotenoids, and the specifically expressed GGPS in leaves is involved in the synthesis of insect pest-induced volatiles (E,E)-4,8,12-trimethyltrideca-1,3,7,11-tetraene (TMTT) [13]. In addition, terpenoids are induced under abiotic stresses, such as UV-B rays, gamma rays, high temperature or the production of reactive oxygen species (ROS) [14]. Under a high-temperature environment, *Quercus ilex* uses monoterpenes to scavenge free radicals, ROS, etc., and releases a large number of volatile monoterpenes to reduce tree body temperature [15]. GGPS is not only important for plant growth and development [16] but has also been widely reported in bacteria [17], fungi [18], insects [19] and animals [20].

Cotton is one of the main cash crops in the world [21]. GGPS is a crucial enzyme for the production of gibberellins, carotenoids, chlorophylls, and rubber, which are structurally diverse classes of isoprenoid biosynthetic metabolites produced by GGPP synthase (GGPPS) in plastids [22, 23]. Currently, the GGPS gene family has been identified in a variety of plants, but there is not enough research on this gene family and related biological function analysis in cotton. Therefore, analyzing the evolution and function of the GGPS gene family in cotton is helpful to screen excellent cotton germplasm resources and deepen the understanding of the biological function of the GGPS gene family. In this study, we downloaded the genome data of four cotton species, namely, *Gossypium hirsutum*, *Gossypium arboreum*, *Gossypium raimondii* and *Gossypium barbadense*, from the cotton database and revealed their evolutionary analysis, cis-acting elements, gene structure, conserved motifs, chromosome location, protein structure and other information through a series of bioinformatics methods. This study provides a theoretical reference for revealing the regulatory mechanism of genetic evolution, growth and chlorophyll synthesis of this gene family in cotton.

## Results

### Identification and sequence analysis of the GGPS gene family in cotton

**Fig. 1:**
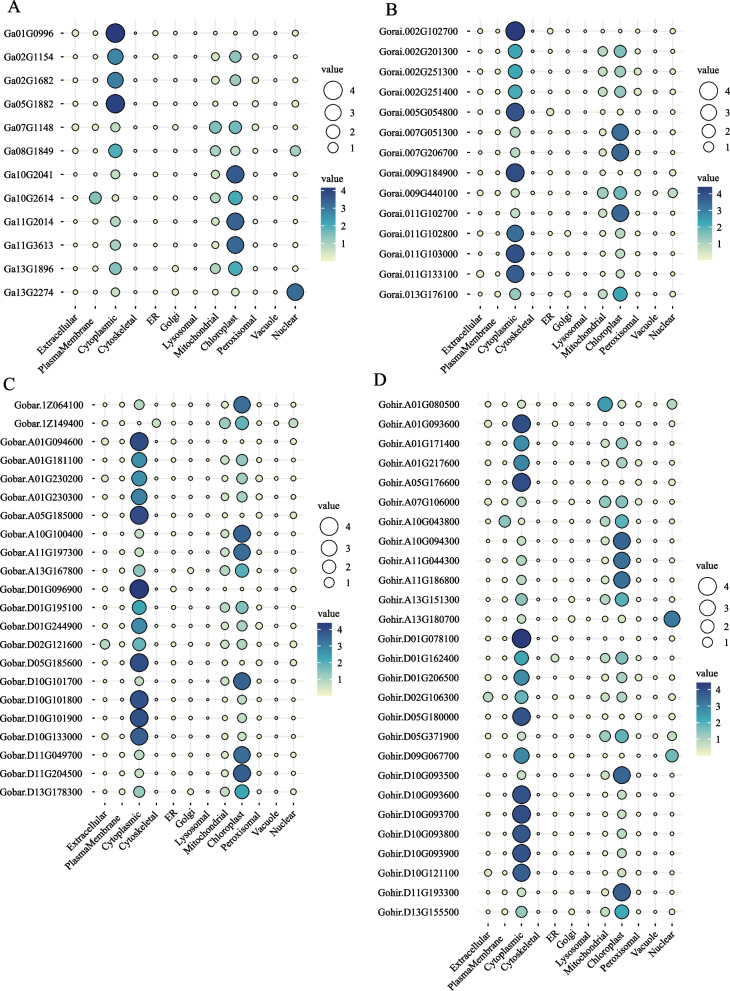
Prediction of the subcellular localization of GGPS genes in *G. arboreum* (**A**), *G. raimondii* (**B**), *G. barbadense* (**C**) and *G. hirsutum* (**D**). The color and the size of the circle indicate the values of the reliable index of the prediction results

Here, we identified 75 GGPS genes in four cotton species from the cottonFGD and Phytozome databases. There were 14, 12, 22 and 27 genes in *G. raimondii*, *G. arboreum*, *G. barbadense* and *G. hirsutum*, respectively. Then, the physiochemical properties and sequences of the members of the GGPS gene family were analyzed (Table S1). The protein molecular weights of GGPS genes were between 14,371.5 ~ 46,093.3 Da, and the average protein molecular weight was 36,159.25. All identified GGPS genes encoded amino acids ranging from 131 to 421, with an average amino acid length of 329.08. The theoretical isoelectric point of these proteins ranged from 4.22 to 7.84, and the average isoelectric point was 5.94, which was weakly acidic. To understand the expression location of the family, the subcellular localization was predicted (Fig. 1). The results showed that almost all GGPS proteins were expressed in the chloroplast, mitochondria and cytoplasm. It was suggested that GGPS family members play different functions in different cell parts. For example, members of the GGPS family located in chloroplasts might play an important role in chloroplast photosynthesis.

### Analysis of the phylogenetic relationship of the GGPS gene family

**Fig. 2:**
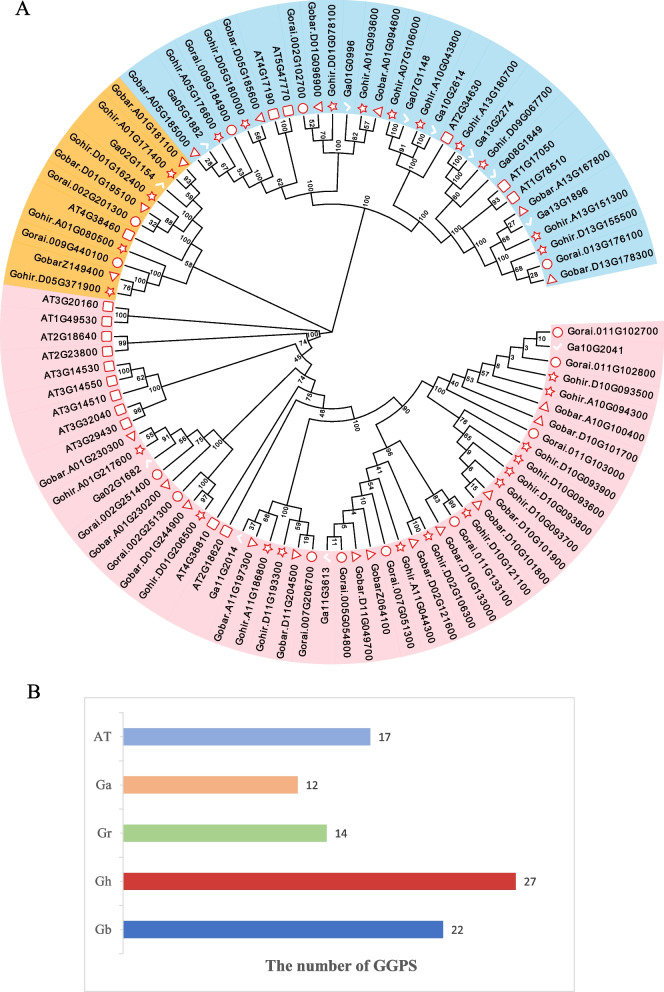
Number of phylogenetic relationships of GGPS family genes in Arabidopsis, *G. hirsutum*, *G. barbadense*, *G. arboreum*, and *G. raimondii*. **A** Phylogenetic tree of the GGPS gene family in four Gossypium species and Arabidopsis. **B** Comparisons of GGPS gene numbers in *A. thaliana*, *G. hirsutum*, *G. barbadense*, *G. arboreum*, and *G. raimondii*. At: *Arabidopsis thaliana*; Ga: *Gossypium arboreum*; Gr: *Gossypium raimondii*; Gb: *Gossypium barbadense*; Gh: *Gossypium hirsutum*

To study the evolutionary relationship among GGPS genes, a phylogenetic tree was constructed using GGPS protein sequences of *A. thaliana*, *G. arboreum*, *G. raimondii*, *G. barbadense* and *G. hirsutum* (Fig. 2). The GGPS genes were divided into three subfamilies; the largest branch contained 39 members of the GGPS family in cotton, and the other two branches contained 10 and 26 GGPS family members. It has been speculated that there is a more advanced evolutionary relationship and similar functions for members of the same branch. According to the phylogenetic tree, most orthologous genes between allotetraploids and diploids are clustered closely to each other in the same group, showing expansion of the GGPS gene family in cotton.

Using the protein sequences, selection pressure was calculated via Calculator 2.0, and the corresponding Ka/Ks values of most of the genes in this family were much less than 1 (Table S2). The rate of synonymous substitution of bases in the development and evolution of most GGPS genes was much higher than that of nonsynonymous substitution, so it was not affected by natural selection. We believe that these genes underwent purifying selection during evolution. There are also some genes with Ka/Ks values greater than 1, such as *Gohir.A10G094300* and *Gohir.D10G093500*, *Gohir.D10G093700* and *Gohir.D10G093900*, *Gohir.D10G093700* and *Gohir.D10G093800*, *Gohir.A10G094300* and *Gohir.D10G093800*, *Gohir.D10G093800* and *Gohir.D10G093900*, *Gobar.D10G101700* and *Gobar.A10G100400*, and *Gorai.011G103000* and *Gorai.011G102700,* indicating that these genes have been positively selected in genetic evolution.

### Gene structure and conserved motif analysis of GGPS proteins

**Fig. 3:**
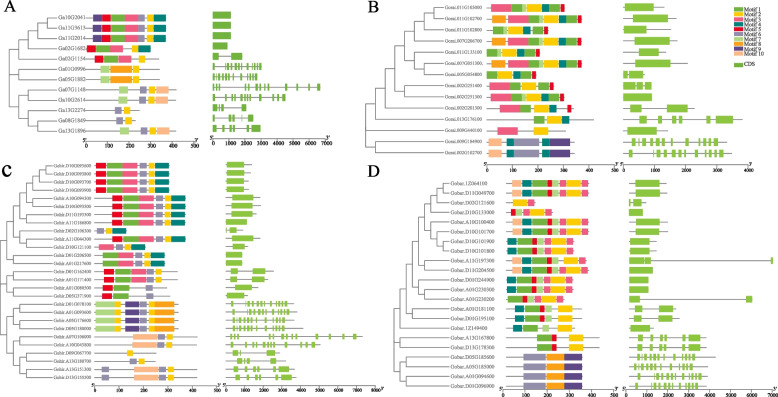
Phylogenetic tree, conserved motifs and gene structure analysis of the GGPS family in *G. arboreum* (**A**), *G. raimondii* (**B**), *G. hirsutum* (**C**) and *G. barbadense* (**D**). Note: The phylogenetic tree was constructed with MEGA 7 using the neighbor–joining (NJ) method with 1000 bootstrap replicates. The conserved motifs in the GGPS proteins were identified with MEME software. Gray lines denote the nonconserved sequences, and each motif is indicated by a colored box. The lengths of motifs in each protein are presented proportionally. The exon‒intron structures of the GGPS genes are based on the evolutionary relationships. The green rectangle represents exons, and the gray line represents introns

To better understand the evolutionary relationship between different members of the GGPS gene family, we constructed phylogenetic trees using the GGPS protein sequence with the NJ method (Fig. 3) and compared and analyzed the intron‒exon structures and conserved motifs of GGPS members of different cotton species. The introns of GGPS genes were different; some GGPS members did not contain introns, while some GGPS genes contained at most 14 introns. The diversity of gene structure indicated that GGPS may have different selection events in the process of gene evolution. Among the four cotton species, the closely related genes in the evolutionary tree tended to have more similar exon and intron arrangements, indicating that the exon‒intron structure was highly related to the phylogenetic relationship between GGPS genes.

Conserved motifs are often related to the function of proteins. To reveal the characteristic motifs of GGPS, the conserved motifs in GGPS proteins were identified by MEME software. A total of 10 conserved motifs were identified, named Motif1 to Motif10, and the number of conserved motifs in each GGPS varied from 1 to 8 (Fig. 3). Deletions of different motifs were found in all 75 members of the GGPS family, but all GGPS genes had a conservative motif distribution pattern, e.g., motif 2 was found in all proteins, indicating that it was highly conserved in GGPS. In summary, upon analysis of the evolutionary tree, gene structure and conserved motifs, it was found that the GGPS members located in the same branch of the evolutionary tree contain similar gene structures and that the composition and arrangement of their conserved motifs are the same. We speculated that these proteins with similar gene structures and motifs may share similar functions and play similar roles in cotton.

### Location and collinearity analysis of GGPS genes on chromosomes

**Fig. 4:**
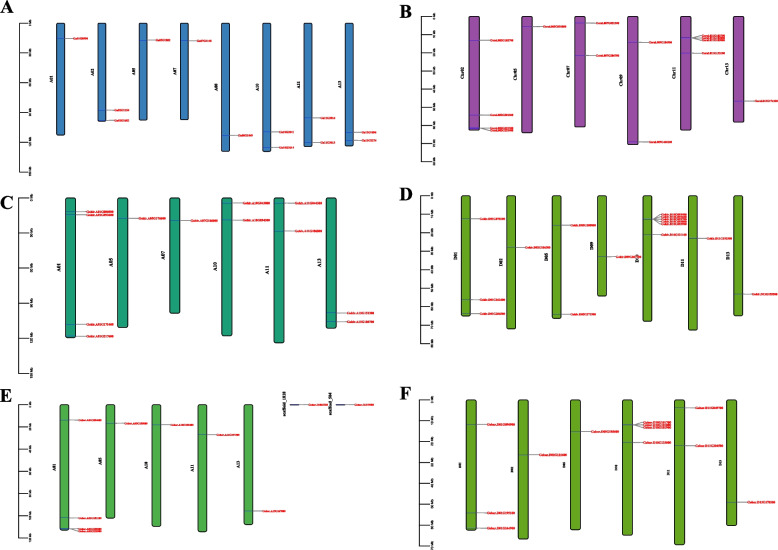
Chromosome distributions of GGPS in *G. arboreum* (**A**), *G. raimondii* (**B**), *G. hirsutum* in At subgenome (**C**), *G. hirsutum* in Dt subgenome (**D**), *G. barbadense* in At subgenome (**E**) and *G. barbadense* in Dt subgenome (**F**) in cotton

The location distribution map of GGPS on the chromosomes of four cotton species was drawn using TBtools software (Fig. 4). The results showed that among the 27 members of the GGPS family in *G. hirsutum*, 12 genes were distributed on 6 chromosomes of the At subgenome, which were A01, A05, A07, A10, A11, and A13, and the other 15 genes were distributed on 7 chromosomes of the Dt subgenome, which were D01, D02, D05, D09, D10, D11 and D13. There were 6 pairs of homologous chromosomes on a total of 13 chromosome pairs. In the genome of *G. barbadense*, we also found that the GGPS gene has a similar distribution on chromosomes. Among the 22 members of the GGPS gene of *G. barbadense*, 8 were distributed on chromosomes A01, A05, A10, A11, and A13 of the At subgenome, and 14 were distributed on chromosomes D01, D02, D05, D10, D11, and D13 of the Dt subgenome. Thus, the genomes of *G. hirsutum* and *G. barbadense* may have come from the same ancestor, and the GGPS gene family is relatively conserved in evolution.

**Fig. 5:**
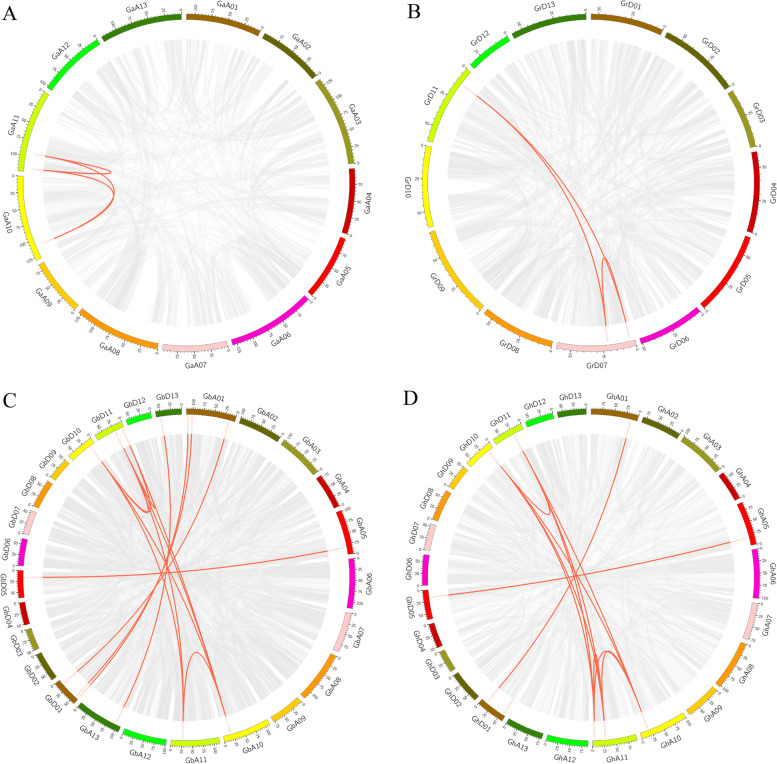
Collinear analysis of the GGPS gene iin *G. arboreum* (**A**). *G. raimondii* (**B**), *G. barbadense* (**C**) and *G. hirsutum* (**D**). Note: Collinear analysis of GGPS genes. The gray lines represent collinear relationships within different genomes, and the red lines represent collinear gene pairs in the GGPS family

In the diploid cotton *G. arboreum*, 12 GGPS genes were distributed on chromosomes A01, A02, A05, A07, A08, A11, and A13. In *G. raimondii*, which is also a diploid species, 14 GGPS members were distributed on chromosomes D02, D05, D07, D09, D11, and D13. Collinearity analysis can well explain the homology between genes, and the collinear homologous sequences may have similar functions, so the collinearity of the GGPS gene family in four different cotton species was analyzed and plotted by MCScanX and Circos software (Fig. 5). We found that the collinearity of GGPS genes in *G. raimondii* mainly occurred between chromosomes D07 and D11 (Fig. 5B). In the *G. arboreum* genome, the collinear region of GGPS genes was between chromosomes A10 and A11 (Fig. 5A). In tetraploid cotton species of *G. hirsutum* and *G. barbadense*, the collinear relationship between genes mostly occurred between homologous chromosomes (Fig. 5C-D). At the same time, there was a collinear relationship between chromosomes A10 and A11 in the two tetraploid cotton species, which was similar to the collinear region of the GGPS family in the *G. arboreum* genome.

### Sequence alignment and three-dimensional structure prediction of GGPS proteins

**Fig. 6:**
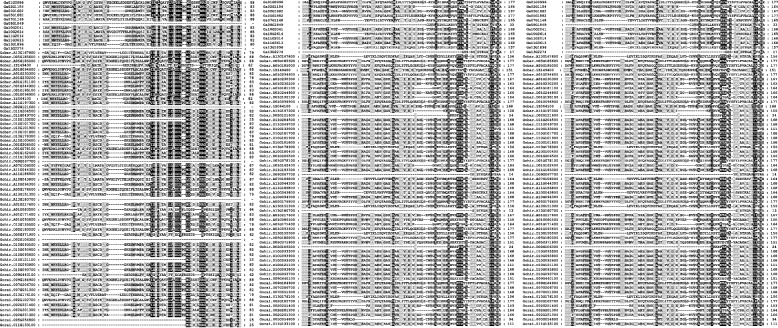
Conserved domains of the GGPS gene family. The black background represents conserved amino acids, and the gray background represents less conserved amino acids

**Fig. 7:**
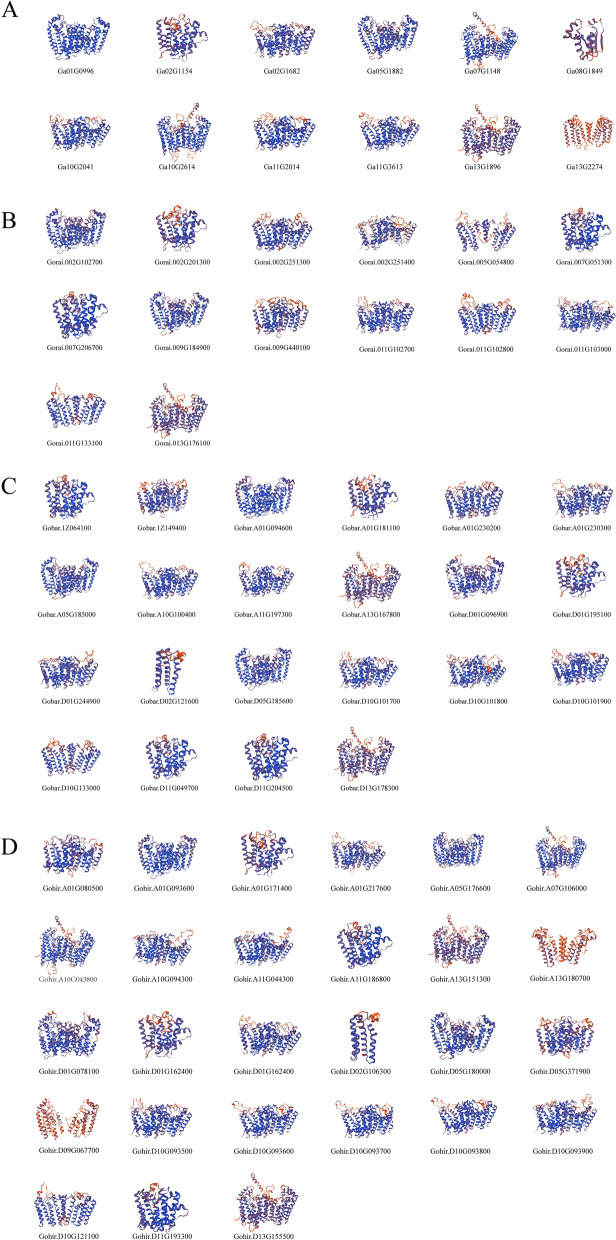
Prediction of the three-dimensional structure of GGPS proteins. (**A**), *G. arboreum *(**B**), *G. raimondii *(**C**), *G. barbadense *(**D**) *G. hirsutum*

The GGPS family is a kind of polypentene synthase in plants. To further determine the sequence characteristics of the cotton GGPS domain, 75 members of the GGPS family were selected for protein sequence alignment and analysis (Fig. 6). The alignment results showed that 75 members of the GGPS family contain two aspartic acid-rich domains: DdxxxxD and DDxxD (x is an arbitrary amino acid), which are typical polypentene synthase domains and beneficial to the binding of IPP and DMAPP and the substrate of GGPS and determine the catalytic activity of GGPS [24].However, a small number of GGPS members had different degrees of deletion of this domain, which might lead to changes in the biological function of these genes. The conformation of proteins is often related to their function. To further understand the function of cotton GGPS proteins, their three-dimensional (3D) structures were predicted through the SWISS-MODEL website (Fig. 7). The results showed that 75 GGPS proteins were mainly composed of α-helices and random crimping, and there was no β-folding. The α-helix is a large number of structural elements in the GGPS polypeptide chain and is scattered in the whole peptide chain. According to protein sequence alignment, it was found that the two functional domains were located in random coils. In addition, there are also some proteins whose 3D structure is too different, such as *Gohir.D02G106300*. This might be due to the differentiation of these genes in evolution, but the 3D structures of the other members were similar, and similar structures often had similar functions. In addition, GGPS is relatively conserved in the process of evolution.

### Analysis of cis-acting elements of GGPS genes in cotton

**Fig. 8:**
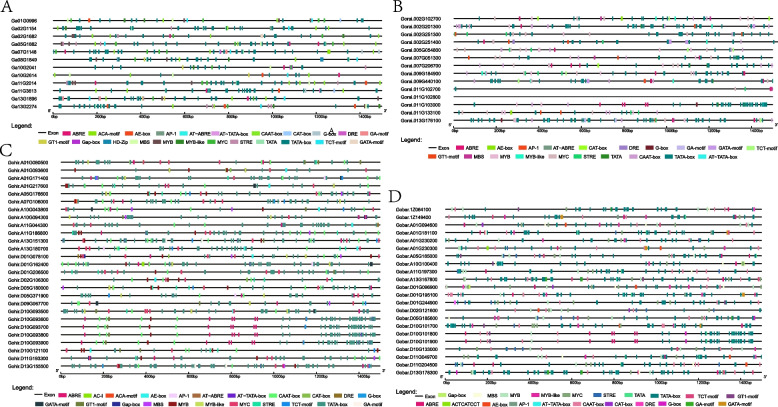
Analysis of cis-acting elements in the 1.5 kb promoter regions of GGPS genes. (**A**), *G. arboreum *(**B**), *G. raimondii *(**C**), *G. hirsutum *(**D**), *G. barbadense*

To understand the potential function of the GGPS gene family, the promoter sequences 1500 bp upstream of GGPS genes were analyzed to detect cis-acting elements (Fig. 8). The results showed that there were many cis-acting elements involved in the physiological process in the upstream promoter region of GGPS genes. There were a large number of cis-acting elements related to light reactions, such as the GA-motif, G-box, TCT-motif, GATA-motif, and GT1-motif. The GGPS gene family may play an important role in the photosynthetic pathway. Of course, there were also cis-acting elements related to abiotic stress responses, such as MYB, ABRE and MBS, in the upstream promoter of the GGPS gene. These results suggested that GGPS genes may also be involved in light response and other physiological processes of biotic and abiotic stresses.

### Virus-induced GGPS gene silencing leads to albinism in leaves

**Fig. 9:**
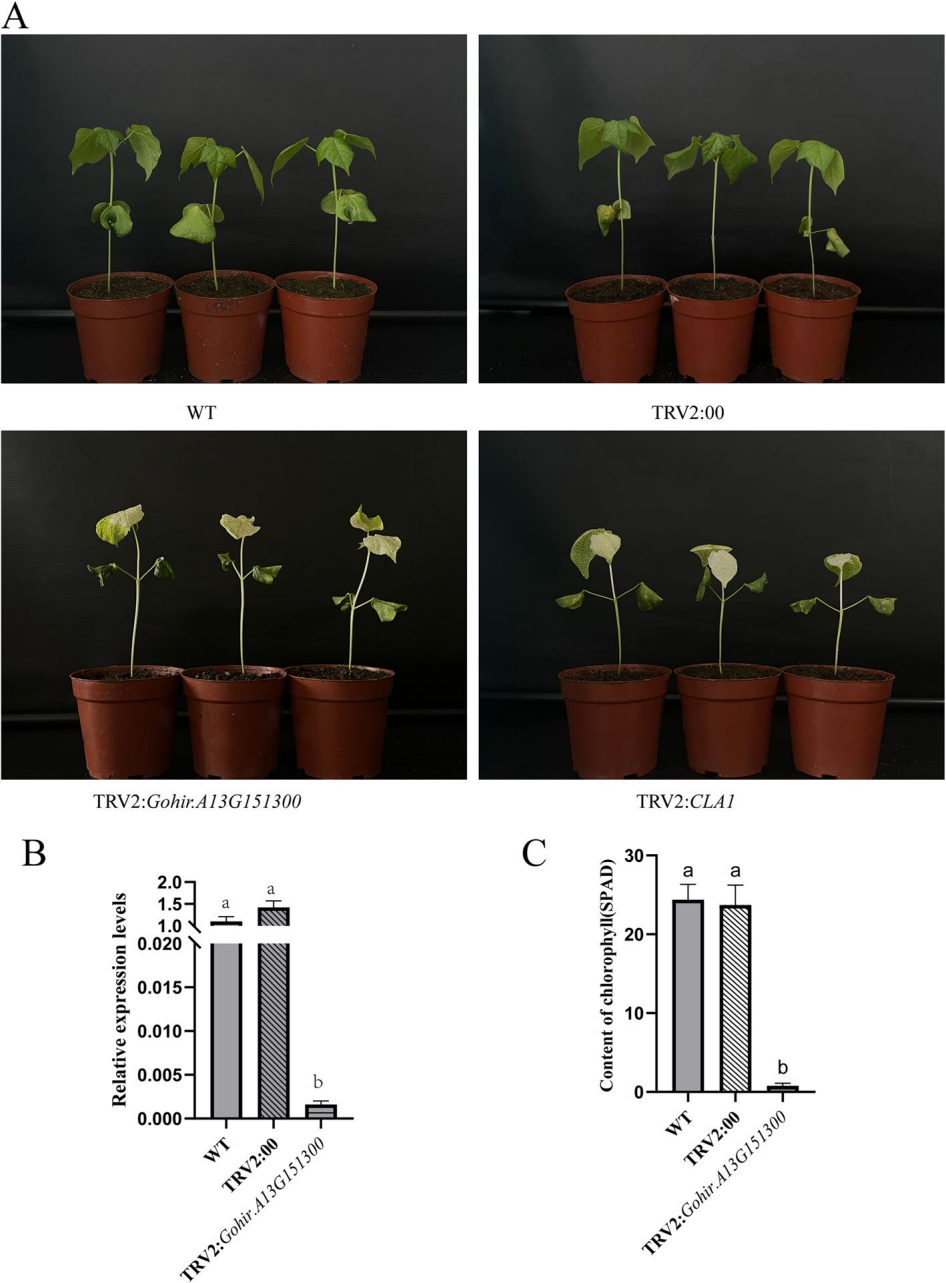
Virus-induced silencing of the GGPS gene in *G. hirsutum.* **A** Phenotypic changes in normal culture of TRV2: *Gohir.A13G151300* and control plants after three weeks of injection. **B** Relative *Gohir.A13G151300* transcript levels in leaves of TRV2: *Gohir.A13G151300* and control plants (TRV2:00). **C** Detection of chlorophyll content in leaves of Wildtype, TRV2:00 and TRV2:*Gohir.A13G151300* plants. Note: Error bars are the means of three replicates ± SD; T test was used for significance test

Virus-induced gene silencing is an effective means to study gene function. To explore the role of GGPS family members in the growth and development of cotton, a VIGS vector was constructed to silence *Gohir. A13G151300* in *G. hirsutum* "CRI 12" (Fig. 9A). After approximately 2 weeks of *Agrobacterium tumefaciens* infection, the new true leaves of TRV2:*CLA1* showed an albino phenotype. This shows that the VIGS program is correct and effective. Then, we took leaf samples and extracted RNA from TRV2:*Gohir. A13G151300* leaves to detect the silencing efficiency. When we compared the silenced gene and TRV2:00 vector as a control, the expression level of this gene was significantly suppressed in TRV2:*Gohir.A13G151300* plants, indicating that it was silenced successfully (Fig. 9B). We found that the plant growth of TRV2:*Gohir. A13G151300* was significantly slower than that of the WT and TRV2:00, and we also found leaf whitening in TRV2:*Gohir.A13G151300*. We measured the relative chlorophyll content of the WT, TRV:00 and experimental groups. We measured the relative chlorophyll content of WT, TRV2:00 and TRV2:*Gohir.A13G151300*. The results showed that the chlorophyll content of TRV2:*Gohir.A13G151300* plants decreased significantly (Fig. 9C). These results suggested that the *Gohir.A13G151300* gene may be involved in the synthesis of photosynthetic pigments in cotton, and the silencing of the gene leads to damage to the photosynthetic system, which leads to leaf albinism and poor growth.

## Discussion

GGPS is an isoprene pyrophosphate synthase ubiquitous in plants, animals and bacteria. GGPP, synthesized by GGPS, is the precursor of many diterpenes and polyterpenes [1, 3]. GGPP can be used as a substrate to participate in various secondary metabolic pathways, including the synthesis of photosynthetic pigments (chlorophyll and carotenoids) [25]. However, to date, there has been no systematic research or analysis of the GGPS gene family in cotton. Cotton is not only an important fiber crop but also one of the main cash crops in China [26]. With the completion of cotton genome sequencing and the development of plant genetics, we can systematically study the structure, location, function and other information about the cotton GGPS gene family. This paper provides basic biological information for further study of the function of the GGPS gene in cotton.

The formation of the gene family may be due to the whole genome duplication event or polyploidy, which is a large-scale chromosome doubling event that increases the number of all genes in a species at once, resulting in the retention of many chromosome doubling fragments in the genome [27]. Allotetraploid cotton evolved from genomic hybridization and subsequent polyploidy of *G. arboreum* and *G. raimondii* [28].

In this study, we identified the GGPS gene in tetraploid cotton of *G. hirsutum*, *G. barbadense* and their two common diploid ancestors, *G. arboreum* and *G. raimondii* [29]. This can be used to speculate the evolutionary relationship of the GGPS gene family in these cotton species. The number of GGPS genes in tetraploid *G. hirsutum* was approximately the sum of the number of GGPS genes of its two diploid ancestors. This shows that there was neither large-scale amplification nor significant gene loss of the GGPS gene family in *G. hirsutum*. In a similar genome-wide study, 26 genes in *G. hirsutum* and *G. barbadense*, and 14 genes in *G. arboreum* and *G. raimondii* were identified in the PFK gene family[30]. Through the phylogenetic tree analysis of GGPS in the four cotton species, we found that 9 of the 17 GGPS genes found in *A. thaliana*, which are also dicotyledons, were closely related to cotton. This phenomenon might be due to different degrees of gene deletion in *A. thaliana* and cotton after deviating from the common ancestor. Through analysis of the location distribution of a total of 75 GGPS on the chromosomes, we could see that the GGPS gene has a similar distribution pattern on the chromosomes of the same tetraploid *G. hirsutum* and *G. barbadense*, which also proved that *G. hirsutum* and *G. barbadense* come from a common ancestor [50]. Tandem duplication mainly occurs in the chromosome recombination region. Members of the gene family formed by tandem duplication are usually closely arranged on the same chromosome, forming a gene cluster with similar sequences and similar functions [31]. Some GGPS genes with similar distances and similar structures were distributed on chromosome D10 of tetraploid cotton and on chromosome 11 of *G. raimondii*. This phenomenon indicated that tandem repeats of the GGPS gene may have occurred in the region of chromosome D10, forming some genes with similar functions. Segmental duplication usually results in replicated genes that are far apart, even on different chromosomes [32]. In the ABC gene family study, an analysis of gene duplication relationships showed how tandem and segmental duplication played a significant part in the large-scale expansion of genes in cotton species [33].

The results of Ka/Ks and collinear analysis were similar; the collinear region of GGPS in tetraploid cotton was mainly concentrated between homologous chromosomes, and the collinear relationship between A10 and A11 in diploid cotton was still retained in tetraploid cotton. This phenomenon indicates that a large number of chromosome recombination events occurred on the homologous chromosomes of tetraploid cotton and that large segments of chromosomes replicated on chromosomes A10-A11 in *G. arboreum* and D07-D11 in *G. raimondii*. According to the results of Ka/Ks, we also found that the vast majority of genes were subjected to purifying selection in the process of evolution; that is, the base substitution on the coding sequence of these genes did not change the composition of proteins, so they were not affected by natural selection, and the functions of these genes were greatly preserved [34]. At the same time, GGPS genes are conserved in the process of chromosome doubling and evolution.

Fragment duplication is important in evolution because many plants have multiple repetitive chromosomal blocks [35]. In addition, *A. thaliana* has been reported to have experienced two whole genome duplications (WGDs) [36]. The gene duplication analysis of the GGPS gene showed that there was a common ancestor between the A-genome and At subgenome, which was similar to the D-genome and Dt subgenome [28]. Some studies have shown that the cotton *GhAAI* gene family [37] and *GhARF* gene family [38] also exhibit expansion. We concluded that large fragment duplication and genomic polyploidy were the main methods of formation of the cotton GGPS gene family. These findings will improve our understanding of chromosome interactions, information exchange, genetic evolution, etc., in cotton.

Through the analysis of conserved motifs and gene structure members of the GGPS gene family, it was found that GGPS had different degrees of motif loss, and it still had similar motif distribution patterns, especially among GGPS members in the same subfamily. The GGPS genes have been found to contain a variety of total introns. According to reports, introns may play a vital role in the evolution of different species [39]. In the early stages of gene expansion, the introns of some genes were lost over time [40]. When introns are under weak selection pressure, genes without introns may evolve rapidly, while genes with larger or more introns contribute to evolution [41]. Therefore, we speculated that some GGPS genes in cotton gradually lose their introns and gain functional evolution over time. In addition, it has been reported that the molecular weight of the fifth amino acid upstream of the FARM domain can determine the length of the carbon chain of the GGPS catalytic product. For example, when the fifth amino acid is methionine with a higher molecular weight, the catalytic product of GGPS is GGPP, whereas when the fifth amino acid is alanine or serine with a lower molecular weight, the catalytic product of GGPS is GFDP (geranylfarnesyl pyrophosphate) [42]. It has been reported that GGPS from angiosperms also contains a conserved CXXC domain [43], but there are also varying degrees of deletion [44]. We also found this phenomenon in the GGPS protein sequences of cotton, and the deletion of this domain may lead to the differentiation of biological function.

In many identified GGPS genes, the subcellular localization of the GGPS protein also showed obvious differentiation. The prediction demonstrated that nearly all GGPS genes were located in the cytoplasm, mitochondria, and chloroplast. In *A. thaliana*, most *AtGGPS* proteins, including *AtGGPS2* and *AtGGPS6*, are located in chloroplasts or plastids, and some GGPS proteins are located in the endoplasmic reticulum and mitochondria, such as *AtGGPS1* and *AtGGPS*3 [10, 43]. The subcellular localization of GGPS members identified in this study predicted the functional differentiation of GGPS genes. Cis-acting element prediction showed that there were many photoresponsive elements upstream of the cotton GGPS gene promoter. In the process of plant evolution, complex mechanisms can be formed to sense light intensity and other factors and affect a series of developmental events, such as seed germination and seedling formation [45]. G-box is the most clearly studied light response regulatory element, which widely exists in the promoters of light-controlled genes and other environmental factors and has a highly conserved core sequence CACGTG [46]. The promoter fragment containing I-box and G-box was linked to the *A. thaliana* ethanol dehydrogenase gene (alcohol dehydrogenase, ADH), which could have light-induced expression activity in tobacco leaves, and the mutant G-box or I-box could reduce the expression activity of the ADH gene and GUS gene [47]. In addition, there were elements related to abiotic stress and growth and development in GGPS, such as MYB, MBS and ABRE.

Some studies have found that *AtGGPS11* can interact with key terpene synthase pathways, such as phytoene synthase (PSY), solanesyl pyrophosphate synthase 2 (SPS2) and GGR, thus directly affecting the synthesis of related terpenoids [48]. Carotenoids, as important photosynthetic pigments in photosynthesis, are important structural components of light energy absorption and transfer and light reaction centers. Its reduction may lead to albinism in plants. It has been reported that through the overexpression of *IbGGPS* in sweet potato, the genes were upregulated and related to the carotenoid pathway and increased the carotenoid content in transgenic plants. The *IbGGPS* gene has the potential to increase the content of carotenoids in sweet potato and other plants [12]. Through VIGS, we silenced *Gohir. A13G151300* in the GGPS family effectively and observed the growth and development of the plant. The leaves were whitened, and the chlorophyll content decreased significantly three weeks after injection. We speculate that the decrease in GGPS content may lead to a decrease in octahydrolycopene production based on GGPP, which leads to a decrease in carotenoid content. In summary, GGPS genes in cotton play an important role in plant carotenoid biosynthesis, but the specific mechanism needs further study.

## Conclusions

Globally, cotton production is a significant economic source of natural fibers. A genome-wide study, RT-qPCR profiling and gene silencing were performed to characterize geranylgeranyl pyrophosphate synthase (GGPS) genes and their role in the growth and development of cotton. In total, 75 genes were identified in four *Gossypium* species, *G. arboreum*, *G. raimondii*, *G. hirsutum*, and *G. barbadense*. Gene knockdown of *Gohir.A13G151300* gene from *G. hirsutum* showed a significant change in the phenotype and physiology of cotton seedlings. The results revealed the formation of albino leaves and a significant reduction in chlorophyll content in the silenced seedlings, suggesting that GGPS genes play an important regulatory role in photosynthesis. This study elucidates information on the role of the GGPS gene family in the growth and development of cotton.

## Materials and methods

### Identification of GGPS gene family members in cotton

The tetraploid cotton species *Gossypium hirsutum* (V2.1) and *Gossypium barbadense* (V1.1) and their common ancestral diploid *Gossypium raimondii* (V2.1) genome databases, including the CDS, gene annotation file and protein sequences, were downloaded from the Phytozome V13 database (https://phytozome-next.jgi.doe.gov/) [49–51]. The *Gossypium arboreum* CRI genome and protein sequences were obtained from the CottonFGD genome database (https://cottonfgd.net/) [52]. The *Arabidopsis* genome and protein sequences were downloaded from the Ensemble website (http://plants.ensembl.org/index.html) [53]. The Pfam number (PF00348) of the GGPS gene family was searched by the Pfam database (http://pfam-legacy.xfam.org/) [54], and the hidden Markov model (HMM) of the GGPS gene family was downloaded. The sequences containing GGPS protein domains in the protein files of four cotton species were searched by HMMER3.0 software and the BLASTP alignment program. The E-value was set to 1e-20 to screen the candidate protein sequence. The candidate protein sequences were uploaded to the SMART database (http://smart.embl.de/) [55], Pfam database (http://pfam-legacy.xfam.org/) and CDD search in the NCBI database (https://www.ncbi.nlm.nih.gov/cdd/) [56] for reidentification. The amino acid sequences of all members of the GGPS gene family of four cotton species were analyzed by ExPASy proteomics Server software (http://www.expasy.org) [57], and the amino acid length and isoelectric point (PI) were calculated. In addition, the subcellular localization of GGPS family members was predicted by the online website WoLF PSORT (https://wolfpsort.hgc.jp/) [58].

### Evolution analysis of GGPS in cotton

To further analyze the relationship between the evolution of the GGPS protein, a phylogenetic tree was constructed by the neighbor-joining method through multiple sequence alignment of the obtained sequences by MEGA7 software [59]. Then, the online website Evolview (https://evolgenius.info//evolview-v2/#login) [60] was used to further modify and beautify the evolutionary tree. We also constructed a local index for the *G. hirsutum* genomic gene sequence and compared the whole CDS data with Blastp through the Blastall program, and the E-value was 1e-20 to obtain the result of *G. hirsutum* genome alignment. Then, the synonymous substitution rate (Ks) and nonsynonymous substitution rate (Ka) of the GGPS gene in cotton were calculated by Calculator 2.0 [61] to analyze gene selection upon evolutionary development.

### Analysis of GGPS family gene structure and motifs in four cotton species

The conserved motifs of GGPS family members were analyzed by using the MEME website (http://meme-suite.org/) [62]. The parameters were set to search the total number of motifs to 10, with the shortest motif length of 6 base pairs and the longest motif length of 50. To analyze the structural information of the GGPS gene, the exon and CDS, 3-UTR, and 5-UTR position information of the GGPS gene on the chromosome was extracted. Then, the structural information of the GGPS gene family was analyzed, and the gene structure map was drawn using the online website GSDS (http://gsds.gao-lab.org/) [63]. Finally, TBtools software was used to combine and visualize the GGPS phylogenetic tree, gene structure and conserved motif images.

### Chromosome mapping and collinearity analysis of the GGPS gene family

The location of GGPS genes on chromosomes was obtained from four kinds of cotton gene annotation files, and then the gene chromosome location map was drawn by Mapchart [64]. By comparing the sequences of all GGPS proteins, the repeatability and collinearity of GGPS proteins in the cotton genome were determined and analyzed by Multiple Collinearity Scan toolkit (MCSCANX) [65] software.

### Cis-acting element analysis

To explore the related functions of gene expression regulation, the promoter sequences 1500 bp upstream of the start codon were obtained from the *G. hirsutum* genome file, and the cis-acting elements of the genes were analyzed. We identified and analyzed the cis-acting elements of the genes by using the PlantCARE database (http://bioinformatics.psb.ugent.be/webtools/plantcare/html/) [66], and the results were mapped using the GSDS online website (http://gsds.gao-lab.org/).

### Multi-sequence alignment and three‑dimensional prediction of the protein structure

To analyze the conserved domain of GGPS proteins, ClustalW from MEGA7 [67] was used for multi-sequence alignment of all protein sequences, and then the conserved sequences of the GGPS gene family were calculated and analyzed by GeneDoc software. To further analyze the protein structure of the GGPS gene family, the 3D structure was predicted according to the GGPS protein sequence. The 3D protein models were constructed on the SWISS-MODEL website (https://swissmodel.expasy.org/) [68] using the homologous protein modeling method.

### Virus-induced gene silencing (VIGS)

The upland cotton line “ CRI12” was selected for VIGS material. Full and similar seeds were soaked in dilute hydrochloric acid, sterilized, and then planted in flowerpots mixed with nutritious soil and vermiculite at 3:1. The temperature of the greenhouse was 25 °C and the light-to-dark ratio was 16 h: 8 h. The VIGS experiment was carried out when the cotyledons of cotton were completely flattened and the first true leaves of cotton had just appeared. The primers designed by Primer Premier 5 were used for the VIGS reaction and ligated to the pTRV2 vector to obtain the recombinant expression vector. A gene *Gohir.A13G151300* was transformed by forward (GCCTCCATGGGGATCCCAAAGTTGTAGCCGATGACC) and reverse (CGAGACGCGTGAGCTCTGCCTGCTTAATCTCACCAC) primer sequences into the pTRV vector using the enzymes Sacl and BamHI to develop pTRV2: *Gohir.A13G151300*. Then, the plasmid was transformed into *Agrobacterium tumefaciens* (GV3101). After screening the positive clones, the bacterial solution was injected into the leaves of cotton seedlings with a sterile syringe.

### Chlorophyll content determination and RT-qPCR analysis

After the appearance of the albino phenotype in the positive seedling stage, the leaves from the same part of cotton were collected to measure the relative content of chlorophyll and extract total RNA. Chlorophyll content was measured using a SPAD meter at the three true leaf stages. We took the average of three measurements as one replication from a single seedling and with a total of three biological replications. Wild-type and pTRV2:00 were used as negative controls while the *Cloroplastos alterados 1* gene (*CLA1*) was used as a positive control.

A ChamQ SYBR qPCR Master Mix (LowROX Premixed) kit was used for real-time quantitative PCR analysis. Primer Premier 5 was used to design RT-qPCR primers for the GGPS gene family. The reaction volume was 20 µL, and the amplification procedures were as follows: pre-denaturation at 95 °C for 30 s, denaturation at 95 °C for 10 s, annealing at 60 °C for 30 s, and 40 cycles [69]. Each set was replicated three times biologically and technically. *Histidine 3* was used as a control, the relative gene expression levels were quantified by 2^−ΔΔCt^, and the significance was tested by T test [70].

## Supplementary Information

**Additional file 1: Supplementary Table S1.** Data for analysis of the physicochemical properties of GGPS family genes of the four cotton species.

**Additional file 2: Supplementary Table S2.** Ka/Ks analysis of the homologous GGPS gene family.

**Additional file 3: Supplementary Table S3.** List of genes and their accession IDs for *G. hirsutum*, *G. arboreum*, *G. raimondii*, *G. barbadense* and *A. thaliana*.

## Data Availability

All the data generated in the study are available publicly in the Phytozome database of the *Gossypium hirsutum* v2.1 genome BioProject, accession numbers of PRJNA515894 and PRJNA713846.

## Abbreviations

GGPS: Geranylgeranyl pyrophosphate synthase
GGPP: Geranylgeranyl pyrophosphate
RT-qPCR: Quantitative real-time polymerase chain reaction
GSDS: Gene Structure Display Server
MW: Molecular weight
PI: Theoretical isoelectric point
HMM: Hidden Markov model
MCScanX: Multiple Collinearity Scan toolkit
WGD: Whole genome duplication
VIGS: Virus-induced gene silencing
IPP: Isopentenyl pyrophosphate
DMAPP: Dimethyl allyl pyrophosphate
